# Correlation between chest radiographic findings and clinical features in hospitalized children with *Mycoplasma pneumoniae* pneumonia

**DOI:** 10.1371/journal.pone.0219463

**Published:** 2019-08-28

**Authors:** Yeon Jin Cho, Mi Seon Han, Woo Sun Kim, Eun Hwa Choi, Young Hun Choi, Ki Wook Yun, SeungHyun Lee, Jung-Eun Cheon, In-One Kim, Hoan Jong Lee

**Affiliations:** 1 Department of Radiology, Seoul National University College of Medicine, Daehak-ro, Jongno-gu, Seoul, Republic of Korea; 2 Department of Radiology, Seoul National University Hospital, Daehak-ro, Jongno-gu, Seoul, Republic of Korea; 3 Department of Pediatrics, Seoul National University College of Medicine, Daehak-ro, Jongno-gu, Seoul, Republic of Korea; 4 Department of Pediatrics, Seoul National University Hospital, Daehak-ro, Jongno-gu, Seoul, Republic of Korea; 5 Institute of Radiation Medicine, Seoul National University Medical Research Center, Daehak-ro, Jongno-gu, Seoul, Republic of Korea; Michigan Medicine, University of Michigan, UNITED STATES

## Abstract

**Background:**

Radiologic evaluation of children with *Mycoplasma pneumoniae* is important for diagnosis and management.

**Objective:**

To investigate the correlation between chest radiographic findings and the clinical features in children with *Mycoplasma pneumoniae* pneumonia.

**Materials and methods:**

This study included 393 hospitalized children diagnosed with *M*. *pneumoniae* pneumonia between January 2000 and August 2016. Their clinical features and chest radiographs were reviewed. Radiographic findings were categorized and grouped as *consolidation group* (*lobar or segmental consolidation*) and *non-consolidation group* (*patchy infiltration*, *localized reticulonodular infiltration*, *or parahilar peribronchial infiltration*).

**Results:**

*Lobar or segmental consolidation* (37%) was the most common finding, followed by *parahilar or peribronchial infiltration* (27%), *localized reticulonodular infiltration* (21%) and *patchy infiltration* (15%). The *consolidation group* was more frequently accompanied by pleural effusions (63%), compared to the *non-consolidation group* (16%). Compared with patients in the *non-consolidation group*, those in the *consolidation group* were associated with a significantly higher rate of hypoxia, tachypnea, tachycardia, extrapulmonary manifestations, prolonged fever, and longer periods of anti-mycoplasma therapy and hospitalization. *Lobar or segmental consolidation* was significantly more frequent in children ≥5 years old (44%) compared with children 2–5 years old (34%) and <2 years old (13%). *Parahilar peribronchial infiltration* was significantly more frequent in children <2 years old (56%) compared with children 2–5 years old (32%) and ≥5 years old (18%).

**Conclusion:**

The chest radiographic findings of children with *M*. *pneumoniae* pneumonia correlate well with the clinical features. Consolidative lesions were frequently observed in older children and were associated with more severe clinical features.

## 1. Introduction

*Mycoplasma pneumoniae* is recognized as one of the most important pathogens causing lower respiratory tract infections [1]. The major burden of *M*. *pneumoniae* infection is in community-acquired pneumonia, which mainly affects young children and adolescents [2]. *M*. *pneumoniae* pneumonia accounts for approximately 10% to 40% of community-acquired pneumonia cases in children [2–4].

Chest radiography is frequently performed in children to diagnose pneumonia and assess its extent. In childhood pneumonia, chest radiography continues to be a valuable method of investigation, because radiographic findings are associated with clinical manifestations [5, 6]. Patients with consolidative pneumonia on radiography require more days of respiratory support, have an increased risk of treatment failure, and have a higher case fatality rate than those with other infiltrates [5, 7]. However, although there have been several studies investigating the relationship between radiologic findings in *M*. *pneumoniae* pneumonia and clinical course, most of these partially evaluated the clinical manifestations in a small study population [8–10].

The purpose of this study was to investigate the correlation between chest radiographic findings and the clinical features in children with *Mycoplasma pneumoniae* pneumonia in a large pediatric patient cohort.

## 2. Materials and methods

The Institutional Review Board approved this study, with a waiver of informed consent requirements (IRB No. H-1711-132-901).

### 2.1. Study subjects

This study included hospitalized children and adolescents under 18 years old who were diagnosed with *M*. *pneumoniae* pneumonia at our hospital between January 2000 and August 2016. The diagnosis of *M*. *pneumoniae* pneumonia was made on the basis of the presence of (i) symptoms and signs indicative of pneumonia, including cough, abnormal breath sounds on auscultation, and lung infiltration on chest radiographs; and (ii) a single anti-mycoplasma antibody titer of ≥1:640, a fourfold or greater rise in titers, a positive test result for *M*. *pneumoniae* by PCR, or *M*. *pneumoniae* isolated on culture of respiratory specimens. Children and adolescents in an immunocompromised state or those chronic lung disease were excluded from this study, because the underlying conditions may preclude diagnosing *M*. *pneumoniae* infection by serology and affect the auscultation and chest radiographic findings. We also excluded patients with asthma in this study because *M*. *pneumoniae* infection can exacerbate asthma and it may exaggerate the clinical and radiological presentations of mycoplasma pneumonia [11, 12].

### 2.2. Clinical and laboratory data collection

Medical records of the study population were retrospectively reviewed. Data on the patient’s age at diagnosis, clinical symptoms and signs, admission to the intensive care unit, laboratory examination results including the test results for *M*. *pneumoniae* infection, and the use of anti-mycoplasma antibiotics (macrolides in all patients and quinolones in 49 patients) were collected. Hypoxiawas defined as when the room air pulse oximetry was <90% or the patient was assumed to be hypoxic and receiving supplemental oxygen [13]. Tachypnea was defined as a respiratory rate of >60 breaths per minute for children aged ≤2 months, >50 for children aged 2–12 months, >40 for children aged 1–5 years, and >20 for children aged >5 years. Tachycardia was defined as >205 beats per minute (bpm) for neonates, >180 bpm for infants, >140 bpm for children aged 1–3 years, >120 bpm for children aged 3–5 years, >118 bpm for children aged 5–10 years, and >100 for adolescents older than 10 years of age [14]. Patients were considered febrile when the axillary body temperature was 38°C or above. The auscultation findings were described as crackles, wheezing, or decreased breath sounds. Extrapulmonary manifestations, such as rash, elevated liver enzyme levels, proteinuria, and arthralgia were evaluated during the hospitalization period [15]. Elevation of liver enzyme levels was defined as at least a twofold increase from the baseline level. Proteinuria was diagnosed when patients younger than the age of 2 years had a urine protein/creatinine ratio >0.5 or patients 2 years of age and older had a ratio >0.2.

### 2.3. Chest radiographic findings and categorization

**Fig 1:**
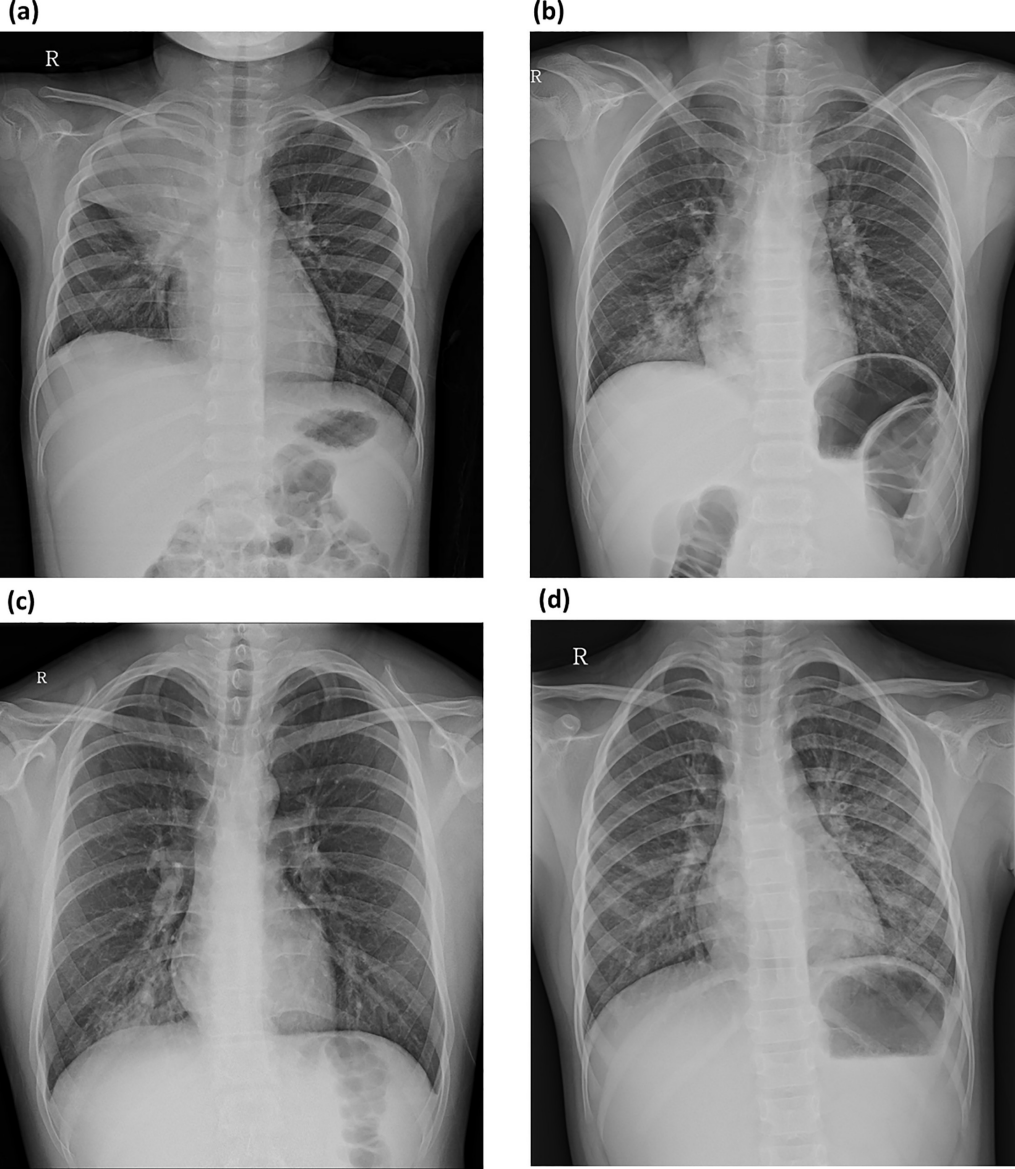
Categorization of the radiographic findings of *M*. *pneumoniae* pneumonia. **(a)** *Lobar or segmental consolidation*. Posteroanterior chest radiograph shows a homogenous dense opacity in the right upper lobe. An air-bronchogram was also noted in the consolidative lesion in the right upper lobe. **(b)** *Patchy infiltration*. Posteroanterior chest radiograph demonstrates localized ill-defined increased lung opacity in the base of the right lower lobe. **(c)** *Localized reticulonodular infiltration*. The chest radiograph shows localized reticulonodular lesions in the base of the right lower lobe. **(d)** *Parahilar peribronchial infiltration*. The chest radiograph demonstrates extensive parahilar reticulonodular lesions in the left upper and lower lung fields.

**Fig 2:**
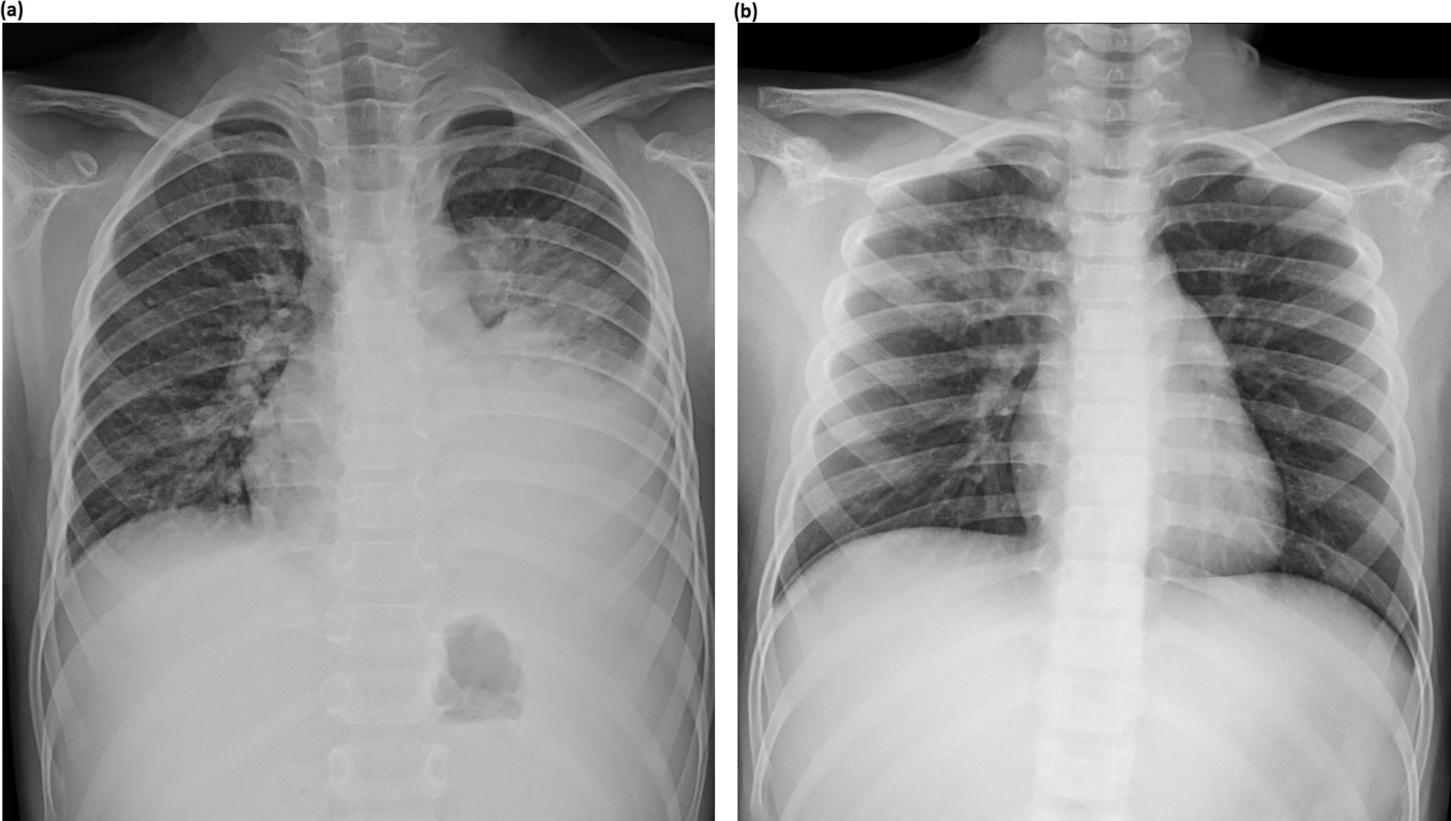
Determination of the dominant radiographic finding. **(a)** A 15-year-old boy with *Mycoplasma pneumoniae* pneumonia. Chest radiograph shows lobar consolidation in the left lower lobe and parahilar peribronchial infiltration in the left lung. In this case, lobar consolidation was regarded as the dominant finding, and categorized as *lobar or segmental consolidation*. **(b)** Chest radiograph of a 9-year-old girl with *Mycoplasma pneumoniae* pneumonia shows both a localized reticulonodular opacity and a patchy opacity in the right upper lung. In this case, the focal reticulonodular opacity was regarded as the dominant finding, and categorized as *localized reticulonodular infiltration*.

The chest radiographs obtained during the course of illness were reviewed by two pediatric radiologists (30-year experience; W.S.K. and 6-year experience; Y.C.) in consensus. The radiologists were unaware of the clinical data associated with each chest radiograph. After analysis of the chest radiograph of each patient, the radiologic findings were categorized as follows: *lobar or segmental consolidation*, *patchy infiltration*, *localized reticulonodular opacity*, and *parahilar peribronchial infiltration* (Fig 1). *Lobar or segmental consolidation* was defined as homogenous dense opacity obscuring pulmonary vascular shadows and involving more than a pulmonary segment. *Patchy infiltration* was defined as a localized ill-defined increased-attenuation lesion or ground-glass lesion involving subsegmental areas. *Localized reticulonodular infiltration* was defined as a reticulonodular opacity involving less than half of one lung field. *Parahilar peribronchial infiltration* was defined as an extensive parahilar reticulonodular lesion involving larger than half of one lung field. For patients with multiple findings in a single chest radiograph, the most dominant finding was chosen (Fig 2). When patients had multiple chest radiographs taken, the radiograph showing the severest features was reviewed. The presence of accompanying pleural effusion was also reviewed. For further analysis, the radiologic findings were grouped as *consolidation group* (*lobar or segmental consolidation*) and *non-consolidation group* (*patchy infiltration*, *localized reticulonodular infiltration or parahilar peribronchial infiltration*).

### 2.4. Chest radiographic findings and the age of patients

We analyzed the age distribution of patients in the *consolidation group* and those in the *non-consolidation group*. To investigate the difference in radiographic findings according to the age of the children, patients were divided into three age groups (<2 years, 2–5 years, and ≥5 years) and their chest radiographic findings were compared.

### 2.5. Diagnostic tests for *Mycoplasma pneumoniae*

Determining the presence of specific antibodies against *M*. *pneumoniae* was performed using an indirect particle agglutination test kit (SerodiaMycoII, Fujirebio, Tokyo, Japan). Antibody titers were tested in dilutions from 1:40 to 1:20,480, and a single titer of ≥1:640 or a fourfold or greater rise in titers was considered suggestive of *M*. *pneumoniae* infection, with reference to previously published data [16]. PCR analysis was performed on nasopharyngeal aspirate specimens that were obtained using mucus traps and catheters within 1–2 days after the patients’ visit to the hospital.

### 2.6. Statistical analysis

The chi-squared test or Fisher’s exact test was used to compare categorical variables, and the Mann-Whitney U test was used to compare continuous variables. Differences were considered statistically significant when the *P* value was below 0.05. SPSS version 23.0 was used to perform the statistical analysis.

## 3. Results

### 3.1. Clinical characteristics of *M*. *pneumoniae* pneumonia

A total of 393 patients were included in this study, and their median age was 5 years (IQR, 3–7 years). Among them, 381 patients (97%) had fever with a median duration of 10 days (IQR, 7–13 days). Eighty-nine patients (23%) presented with hypoxia and received supplementary oxygen therapy. Sixty-three patients of them had documented hypoxemia on pulse oximetry and remaining 26 patients were assumed to be hypoxic by clinicians. Tachypnea and tachycardia were noted in 63% and 79% of the children, respectively. On auscultation, crackles (53%) were predominantly heard, followed by decreased breath sounds (27%) and wheezing (10%). Extrapulmonary manifestations were observed in 30% of the patients. Liver enzyme level elevation (58%) and rash (55%) were the two most common findings, followed by proteinuria (9%) and arthralgia (8%). Among the patients, 2% were admitted to the intensive care unit due to respiratory failure. Three hundred and forty-six patients (88%) received anti-mycoplasma antibiotic treatment for a median duration of 13 days (IQR, 10–16 days). Beta-lactam antibiotics were co-administered in 183 (47%) patients as empirical therapeutics for other potential bacteria. The median hospitalization period was 6 days (IQR, 2–9 days).

### 3.2. Chest radiographic findings of *M*. *pneumoniae* pneumonia

The most common chest radiograph finding in *M*. *pneumoniae* pneumonia was lobar or segmental consolidation (37%), followed by *parahilar peribronchial infiltration* (27%), *localized reticulonodular infiltration* (21%), and *patchy infiltration* (15%) (Table 1). Multiple findings in a single chest radiograph were observed in 123 of 393 patients (31%). Pleural effusion was detected in 132 of 393 patients (34%) and was more frequently associated with *lobar or segmental consolidation* (63%) and *patchy infiltration* (25%) compared to *localized reticulonodular infiltration* (13%) or *parahilar peribronchial infiltration* (14%). The differences in the distribution of pleural effusion among the four different chest radiographic findings were statistically significant (P < 0.001) except that between the localized *reticulonodular infiltration* and *parahilar peribronchial infiltration* groups. Twelve of the 132 patients (9%) showed massive pleural effusion, requiring percutaneous catheter drainage.

**Table 1:** Chest radiograph findings of *Mycoplasma pneumoniae* pneumonia in children.

	Consolidation group	Non-consolidation group			*P-value*
	*Lobar or Segmental consolidation*	*Patchy* *infiltration*	*Localized reticulonodular infiltration*	*Parahilar peribronchial infiltration*	
Patients, N (%)	146 (37)	57 (15)	83 (21)	107 (27)	
Pleural effusion, N (%)	92 (63)	14 (25)	11 (13)	15 (14)	<0.001*

*P <0.001 for consolidation vs. other infiltrations and also when each one of the four categories were separately compared except for localized reticulonodular opacity vs. parahilar peribronchial infiltration.

### 3.3. Association between chest radiographic findings and clinical manifestations

The difference between the *consolidation group* and the *non-consolidation group* on chest radiographs in relation to clinical manifestations is shown in Table 2. The *consolidation group* on chest radiographs had a longer median fever duration (12 days vs. 9 days, *P* < 0.001) and were more likely to have hypoxia(*P* = 0.006), tachypnea (*P* = 0.001), and tachycardia (*P* = 0.010). The incidence of chest retraction showed no difference between both groups. Breath sounds were decreased in the *consolidation group* (*P* < 0.001), whereas crackles were frequently heard in the *non-consolidation group* (*P* <0.001). Extrapulmonary manifestations, such as rash and liver enzyme level elevation, were more frequently observed in the *consolidation group* (*P* < 0.001). The white blood cell count was higher in the *non-consolidation* than in the *consolidation group* (8.6 x 10^3^/μL vs. 7.7 x 10^3^/μL, respectively, *P* = 0.006). However, the median level of C-reactive protein was higher in the *consolidation* group than in the *non-consolidation group* (5.3 mg/dL vs. 2.6 mg/dL, respectively, *P* < 0.001). In the *consolidation group*, more patients received anti-mycoplasma antibiotics than in the *non-consolidation group* (95% and 84%, respectively, *P* = 0.008), and the *consolidation group* required treatment with anti-mycoplasma antibiotics for a longer duration than the *non-consolidation group* (15 days vs. 11 days, *P* = 0.001). The median hospitalization period was longer in the *consolidation group* than in the *non-consolidation group* (8 days vs. 5 days, *P* < 0.001). There was no difference in the requirement of intensive care unit admission between the two groups. When the four categories of radiologic findings were separately analyzed, children with *lobar or segmental consolidation* showed the longest median fever duration (12 days, *P* < 0.001) as well as the highest incidence of hypoxia (30%, *P* = 0.006), tachypnea (75%, *P* < 0.001), tachycardia (86%, *P* = 0.01), and decreased breath sounds (53%, *P* < 0.001). Extrapulmonary manifestations were most commonly observed in the patients with *lobar or segmental consolidation* (47%, *P* < 0.001). The median level of C-reactive protein was higher in patients with *lobar and segmental consolidation* (5.3 mg/dL, *P* < 0.001) and these patents were treated with anti-mycoplasmal antibiotics and hospitalized for the longest period (15 days, *P* < 0.001) among the four radiologic category groups.

**Table 2:** Association between chest radiograph findings and clinical manifestations in children with *Mycoplasma pneumoniae* pneumonia.

Clinical variables	*Consolidation group* (n = 146)	*Non-consolidation group* (n = 247)	*P-value*
Age, median (IQR) (y)	5 (4–8)	4 (3–7)	<0.001
Clinical Signs			
Fever duration, median (IQR) (d)	12 (9–14)	9 (6–11)	<0.001
Hypoxia	30%	18%	0.006
Tachypnea	75%	56%	<0.001
Tachycardia	86%	74%	0.010
Chest retraction	12%	13%	0.965
Crackles	38%	62%	<0.001
Wheezing	6%	12%	0.071
Decreased breath sound	53%	11%	<0.001
Extrapulmonary manifestation	47%	19%	<0.001
Rash	25%	11%	<0.001
Liver enzyme elevation	32%	9%	<0.001
Laboratory findings			
WBC count, median (IQR) (x10^3^/μL)	7.7 (5.9–10.3)	8.6 (6.5–12.1)	0.006
CRP, median (IQR) (mg/dL)	5.3 (2.1–11.4)	2.6 (0.9–5.0)	<0.001
ICU admission	3%	1%	0.134
Anti-mycoplasmal antibiotics treatment	95%	84%	0.008
Duration, median (IQR) (d)	15 (12–18)	11 (7–14)	<0.001
Hospitalization period, median (IQR) (d)	8 (5–11)	5 (2–7)	<0.001

Values are percentage unless otherwise stated.

Abbreviations: y, year; d, day; IQR, interquartile range; WBC, white blood cell; CRP, C-reactive protein; ICU, intensive care unit

### 3.4. Chest radiographic findings and the age of patients

The median age of the *consolidation group* was higher than that of the *non-consolidation group* (5 years vs. 4 years, *P* < 0.001) (Table 2). The radiographic features according to three different age groups are shown in Table 3. *Lobar or segmental consolidation* was most frequently observed in children aged ≥5 years (44%), followed by children aged 2–5 years (34%) and children aged <2 years (13%); this difference was statistically significant (*P* < 0.001). In comparison, *parahilar peribronchial infiltration* was observed in 56% of children aged <2 years, followed by 32% of children aged 2–5 years and 18% of children aged ≥5 years; this difference among the age groups was statistically significant (*P* < 0.001). There was no difference in the incidence of *patchy infiltration* or *localized reticulonodular infiltration* among the different age groups.

**Table 3:** Chest radiograph findings of children with *Mycoplasma pneumoniae* pneumonia by age.

	<2 years (n = 39)	2–5 years (n = 154)	≥5 years (n = 200)		*P-value*
*Consolidation group*	5 (13%)	52 (34%)	89 (44%)		<0.001*
*Lobar or Subsegmental consolidation*	5 (13%)	52 (34%)	89 (44%)		<0.001*
*Non-consolidation group*	34 (87%)	102 (66%)	111 (56%)		<0.001*
*Patchy infiltration*	5 (13%)	20 (13%)	32 (16%)		0.892
*Localized reticulonodular infiltration*	7 (18%)	32 (21%)	44 (22%)		0.844
*Parahilar peribronchial infiltration*	22 (56%)	50 (32%)	35 (18%)		<0.001*

*P <0.05, <2 years vs. 2–5 years, 2–5 years vs. ≥5 years, and <2 vs. ≥5 years of age.

## 4. Discussion

The chest radiographic examination is an essential part of the diagnosis of pneumonia including *M*. *pneumoniae* pneumonia. Furthermore, chest radiographs play an important role in assessing a patient’s current condition and prognosis, as well as in determining the treatment plan. The radiographic findings of *M*. *pneumoniae* pneumonia in children are nonspecific and variable and may include localized reticulonodular opacities, parahilar peribrochial infiltrations, localized ground glass lesions, lobar consolidation, and mixed interstitial and focal air-space pneumonia at multiple sites [17]. In addition, minimal or small pleural effusions and hilar lymphadenopathy can be associated [17]. Typical radiographic findings of *M*. *pneumoniae* pneumonia were known to be focal reticulonodular opacification or peribronchial interstitial infiltrates [9, 17, 18]. However, several authors have stated that patchy consolidation or homogenous consolidation are common findings in *Mycoplasma pneumoniae* pneumonia [8, 19–21]. In previous studies of pediatric patients, the proportions of patients with consolidative lesions were 33 to 70% [9, 17, 21].

The various and variable radiologic features can be understood through the pathophysiological mechanism of mycoplasma pneumonia. *M*. *pneumoniae* attach to the ciliated epithelial cells on the respiratory tract through the P1 protein, further protecting the organism from mucociliary clearance and producing local cytotoxic effects [22]. Therefore, the characteristic histopathologic features are edematous and ulcerative bronchial and bronchiolar walls that are infiltrated with macrophages, lymphocytes, neutrophils, and plasma cells. These findings manifest as peribronchial infiltrates and nodular opacities on chest radiograph [23]. In severe pneumonia, the cell-mediated immune response is exaggerated, and interleukin levels are elevated, resulting in diffuse alveolar damage with fibrinous exudates within the alveolar lumens, appearing as consolidation on radiographs [24–26]. Several studies already noted that children with severe symptoms were likely to show consolidative features at the radiologic examination [8–10].

In this study, *lobar or segmental consolidation* (37%) was the most common finding in *M*. *pneumoniae* pneumonia. In a previous study with 42 patients which consisted of both outpatients and inpatients with *M*. *pneumoniae* pneumonia, the most common radiological finding was focal reticular opacity (62%). In their study, consolidation or pseudoconsolidation, which also included patchy opacities, were seen in 33% of patients [17]. The subjects of this study consisted of patients who visited and were hospitalized in a tertiary hospital; therefore, patients who had severe symptoms at the beginning of disease or persistent symptoms in spite of treatment at the primary or secondary hospital were more likely to be selected. This could also explain the relatively high incidence (34%) of pleural effusion in this study. The incidence of pleural effusion in *M*. *pneumoniae* pneumonia is estimated to range from 5% to 24% in other previous studies [17, 27, 28]. In this study, the incidence of pleural effusion was high, especially in patients with *lobar or segmental consolidation* (63%).

This study involving a large study population with *M*. *pneumoniae* pneumonia found that the chest radiographic findings correlated well with the clinical manifestations. In several previous studies on hospitalized children with mycoplasma pneumonia, children with lobar or segmental consolidation had a longer fever duration and longer hospitalization period compared with those with non-consolidative lesions [8–10]. In this study, children with *lobar or segmental consolidation* especially, exhibited the longest fever duration with the highest incidence of extrapulmonary manifestations.

In laboratory results, the C-reactive protein level was significantly higher in the *consolidation group* than in the *non-consolidation group*. This correlation between laboratory findings and radiologic findings is in keeping with the findings of previous studies [9, 10]. However, white blood cell (WBC) counts were higher in the non-consolidation group than in the consolidation group, although the clinical significance of this result is unclear. This study further assessed hypoxia, tachypnea, and tachycardia, which indicate severe pneumonia in children, and observed a higher incidence in patients with consolidation [29]. The background of this interrelationship between the symptoms of pneumonia and radiologic findings in *M*. *pneumoniae* pneumonia could be explained by the immune response as well as pathologic changes. As already mentioned, the cell-mediated immune response plays an important role in the development of *M*. *pneumoniae* pneumonia [30]. The excessive cell-mediated immune response of the host not only induces the formation of pulmonary consolidation, but also contributes to severe respiratory manifestations [30]. Considering the high proportion of extrapulmonary manifestations in this study, children with *lobar or segmental consolidation* might have an augmented immune response. From these findings, we could further conjecture that this specific subgroup might benefit from anti-inflammatory therapies besides anti-mycoplasma antibiotics.

Previous studies on *M*. *pneumoniae* pneumonia in children showed that consolidations were more frequently observed in older age, while interstitial changes were most commonly reported in young children [10, 19], which are consistent with the results of this study. In this study, children ≥5 years old were likely to show consolidations (44%), while only 13% of children <2 years old had consolidations. A possible explanation would be the more mature response of T lymphocytes to alveolar macrophages in older children, producing higher levels of cytokines and resulting in more severe pneumonia [26, 31, 32].

This study has several limitations. First, as the study was performed retrospectively, clinical information may have been limited to available medical records of the patients. Second, some radiographic findings are difficult to classify into the four different categories. Third, because this study was performed at a tertiary hospital, many patients had pneumonia severe enough to be referred from primary or secondary hospitals. Therefore, it may be difficult to apply the results of this study to general patient group. Fourth, although we excluded patients with asthma in study population, it is possible that the cases that had not been confirmed as asthma at the time of diagnosis of *M*. *pneumoniae* pneumonia could be included.

## 5. Conclusion

The chest radiographic findings in childhood *M*. *pneumoniae* pneumonia are variable and nonspecific. Common radiographic findings of *M*. *pneumoniae* pneumonia were *lobar or segmental consolidation*, *parahilar peribronchial infiltration*, *localized reticulonodular infiltration*, and *patchy infiltration*. The chest radiographic findings of children with *M*. *pneumoniae* pneumonia correlate well with the clinical features. The *consolidation group* was associated with severe clinical features. *Lobar or segmental consolidation* was more frequently observed in older children. The difference in chest radiographic findings in different age groups may provide guidance to clinicians for managing *M*. *pneumoniae* pneumonia in children.

## Supporting information

S1 File Dataset. Individual patient data of clinical and radiologic findings.
(XLSX)

## Abbreviations

Bpm: Beats per minute
IQR: Interquartile range
CT: Computed tomography
